# 1,2-Bis[di­(benzo­furan-2-yl)phosphan­yl]ethane

**DOI:** 10.1107/S2414314625009162

**Published:** 2025-10-24

**Authors:** Sebastian Ahrens, Anke Spannenberg, Matthias Beller, Kathrin Junge

**Affiliations:** a Leibniz-Institut für Katalyse e V Albert-Einstein-Str 29a 18059 Rostock Germany; Katholieke Universiteit Leuven, Belgium

**Keywords:** crystal structure, bidentate ligand, benzo­furan, phosphine

## Abstract

The title compound, C_34_H_24_O_4_P_2_, consists of an ethyl­ene-bridged diphosphine with benzo­furan residues, where the P—C—C—P backbone exhibits an *anti*-conformation. The asymmetric unit contains one half mol­ecule, which is completed by inversion symmetry.

## Abstract 2

A new electron-rich, bidentate phosphine, 1,2-bis­[di­(benzo­furan-2-yl)phosphan­yl]ethane, was synthesized and structurally characterized by single-crystal X-ray diffraction.

## Graphical abstract

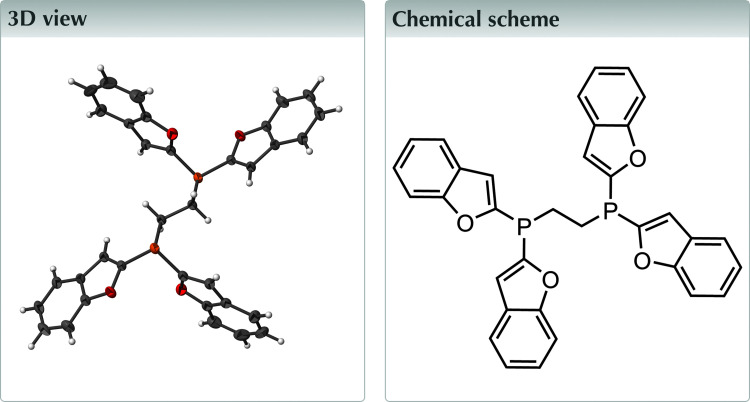

## Structure description

Bidentate phosphine ligands play a pivotal role in homogeneous catalysis, where their chelation to a metal center enhances complex stability and allows precise control over electronic and steric properties (van Leeuwen *et al.*, 2000 ▸). Ethyl­ene-bridged diphos­phines, such as 1,2-bis­(di­phenyl­phosphan­yl)ethane (dppe), are among the most widely used ligands owing to their versatile coordination behavior (Clevenger *et al.*, 2020 ▸). Variations in the substituents on phospho­rus strongly influence catalytic activity, selectivity, and metal–ligand inter­actions. Consequently, structural modification of ethyl­ene-bridged diphosphines remains a key strategy for the design of improved catalysts in transition-metal-mediated transformations. Numerous ethyl­ene-bridged diphosphines have been synthesized, bearing alkyl substituents as well as aryl substituents (Dekker *et al.*, 1992 ▸).

Recently, our group demonstrated the potential of benzo­furan-based phosphines in the Co-catalyzed isomerization of allyl­amines (Ahrens *et al.*, 2025 ▸). These studies revealed that benzofurylphosphines represent an alternative structural motif to conventional aryl phosphines, offering distinct electronic and steric properties that can significantly influence catalytic activity. Owing to their unique reactivity, structurally related phosphines have also been successfully applied in the Pd-catalyzed telomerization of butadiene (Souza *et al.*, 2025 ▸). In our previous work, only monodentate benzo­furan phosphines were developed and evaluated in catalytic applications. To expand this ligand family, the corresponding bidentate analogue has now been synthesized. The new diphosphine 1,2-bis­[di­(benzo­furan-2-yl)phosphan­yl]ethane was prepared and its crystal structure determined.

**Figure 1:**
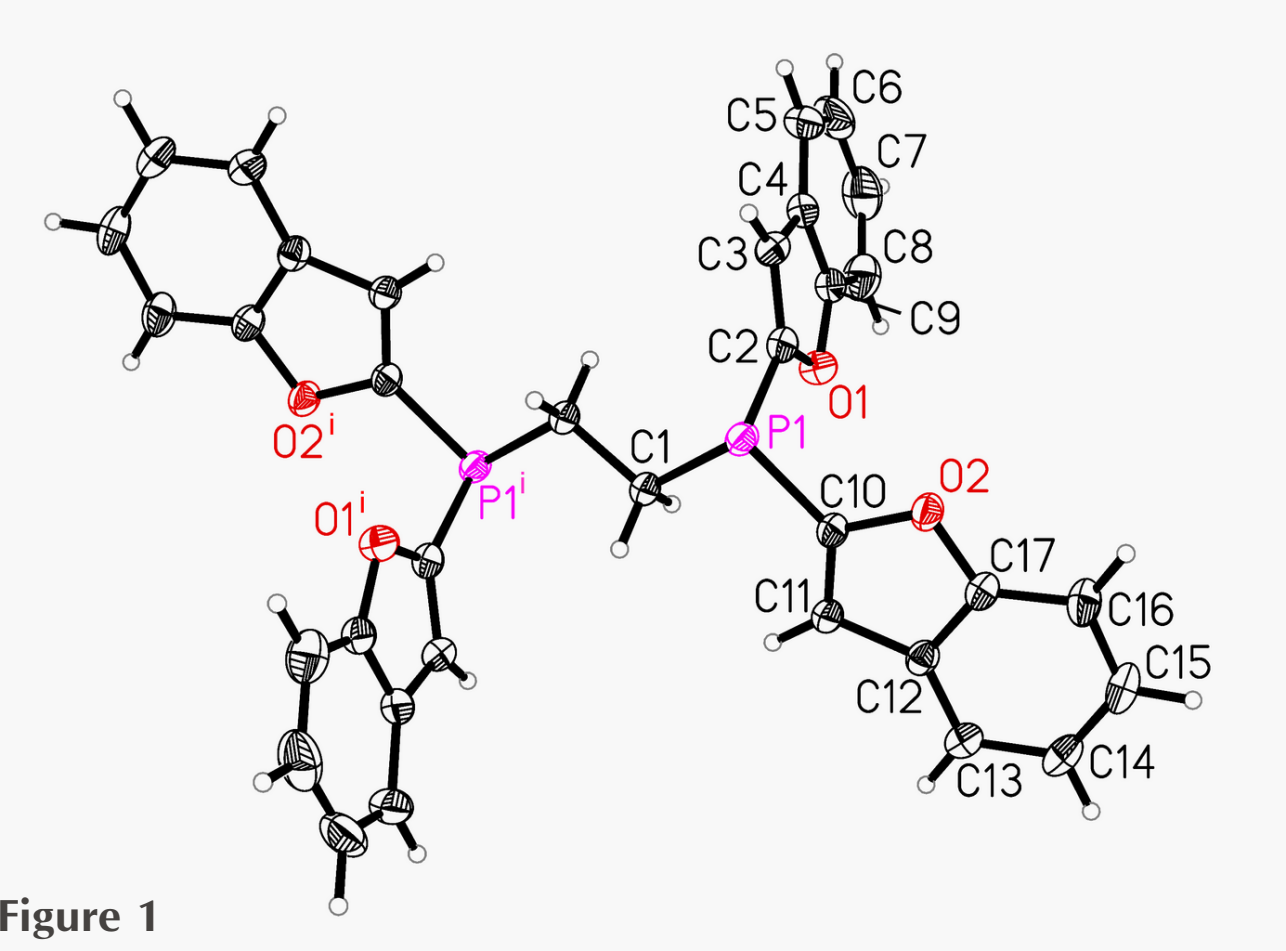
The mol­ecular structure of the title compound with atom labeling and displacement ellipsoids drawn at 50% probability level [symmetry code: (i) −*x* + 1, −*y* + 2, −*z* + 1].

The mol­ecular geometry of the title compound reflects the characteristic features of ethyl­ene-bridged diphosphines with the P–C–C–P backbone forming a zigzag chain and exhibiting an *anti*-conformation (Fig. 1 ▸). Each phospho­rus atom displays a pyramidal arrangement with two benzo­furan substituents and one CH_2_ group of the ethyl­ene bridge. The P—C—C—P torsion angle amounts to 180°, bond lengths and angles are in the expected range. The dihedral angle between the benzofuran rings is 84.94 (3)°. The asymmetric unit contains one half-mol­ecule expanded by the symmetry operation −*x* + 1, −*y* + 2, −*z* + 1.

Moreover, the electronic structure of the benzofuryl diphosphine differs significantly from that of dppe. In solution at room temperature, the ^31^P NMR resonance is significantly upfield-shifted (–52.6 ppm compared to −12.6 ppm for dppe), indicating a higher electron density at the phospho­rus atoms (Benny *et al.*, 2023 ▸). The increased shielding can be attributed to the greater π-donor strength and electron delocalization provided by the benzofuryl substituents. Therefore, the ligand shows an increased electron-donating character, altering the electron density and reactivity of its metal complexes relative to dppe.

## Synthesis and crystallization

All synthetic procedures were carried out under argon atmosphere using standard Schlenk techniques. The anhydrous and oxygen-free solvents used (tetra­hydro­furan, di­chloro­methane, diethyl ether, and *n*-penta­ne) were obtained from an Innovative Technology PS-MD-6 solvent purification system. The purified solvents were stored over 3 Å mol­ecular sieves under argon. The reagents 1,2-bis­(di­chloro­phosphan­yl)ethane and benzo­furan were obtained from Sigma-Aldrich and Fisher Scientific, respectively, and used as received.

NMR spectra were recorded on a Bruker Avance 300 spectrometer operating at 300 MHz for ^1^H, 75 MHz for ^13^C, and 121 MHz for ^31^P. All chemical shifts (δ) are reported in ppm relative to tetra­methyl­silane (TMS). Solvent references for CD_2_Cl_2_ are δ = 5.32 ppm for ^1^H and 53.84 ppm for ^13^C. ^31^P chemical shifts are reported relative to an external 85% H_3_PO_4_ standard.

The synthesis of the title compound was carried out following literature procedures for ethyl­ene-bridged diphosphines with minor modifications (Casey *et al.*, 1983 ▸). Under an argon atmosphere, anhydrous benzo­furan (1.181 g, 10.0 mmol, 4 eq.) was charged in a Schlenk flask and dissolved in 20 ml of anhydrous THF. The solution was cooled to 253 K, and *n*-BuLi (2.5 *M*, 4.0 ml, 10.0 mmol, 4 eq.) was added dropwise. The reaction mixture was stirred for 2 h. Subsequently, 1,2-bis­(di­chloro­phosphan­yl)ethane (580 mg, 2.5 mmol, 1 eq.) was added slowly to the li­thia­ted benzo­furan solution. The reaction temperature was maintained at 253 K for 2 h before allowing the reaction mixture to warm to room temperature. After stirring overnight, the solvent was removed *in vacuo*, yielding a yellow solid. To remove lithium chloride, the yellow solid was dissolved in 60 ml of anhydrous diethyl ether and the resulting suspension was filtered under an inert atmosphere. 1,2-Bis[di­(benzo­furan-2-yl)phosphan­yl]ethane was crystallized from a concentrated di­chloro­methane solution at 278 K to afford colorless, needle-shaped crystals (894 mg, 1.6 mmol, 64%). Crystals suitable for single-crystal X-ray diffraction were obtained by diffusion of *n*-pentane into a di­chloro­methane solution of the phosphine.

**^1^H NMR (300 MHz, CD_2_Cl_2_):** δ = 7.55 (*ddd*, *J* = 7.5, 1.4, 0.7 Hz, 4H), 7.46–7.39 (*m*, 4H), 7.35–7.18 (*m*, 8H), 7.13 (*q*, *J* = 1.0 Hz, 4H), 2.54 (*dd*, *J* = 5.6, 5.0 Hz, 4H).

**^13^C NMR (75 MHz, CD_2_Cl_2_):** δ = 158.17, 154.26 (*dd*, *J* = 11.0, 8.9 Hz), 128.29 (*t*, *J* = 3.2 Hz), 125.68, 123.29, 121.64, 117.50 (*t*, *J* = 15.5 Hz), 111.75, 21.43 (*dd*, *J* = 6.4, 5.0 Hz).

**^13^C-DEPT-135 NMR (75 MHz, CD_2_Cl_2_):** δ = 125.12 (CH, pos.), 122.73 (CH, pos.), 121.08 (CH, pos.), 116.94 (CH, pos.), 111.19 (CH, pos.), 20.77 (CH_2_, neg.).

**^31^P NMR (122 MHz, CD_2_Cl_2_):** δ = −52.60.

**HRMS (ESI):***m*/*z* calculated for C_34_H_24_O_4_P_2_: 558.1150 [M+H]^+^, found: 559.1219.

## Refinement

Crystal data, data collection and structure refinement details are summarized in Table 1 ▸.

## Supplementary Material

Crystal structure: contains datablock(s) I. DOI: 10.1107/S2414314625009162/vm4075sup1.cif

Structure factors: contains datablock(s) I. DOI: 10.1107/S2414314625009162/vm4075Isup2.hkl

Supporting information file. DOI: 10.1107/S2414314625009162/vm4075Isup3.cml

CCDC reference: 2496508

Additional supporting information:  crystallographic information; 3D view; checkCIF report

## Figures and Tables

**Table 1:** Experimental details

Crystal data	
Chemical formula	C_34_H_24_O_4_P_2_
*M*_r_	558.47
Crystal system, space group	Monoclinic, *P*2_1_/*n*
Temperature (K)	150
*a*, *b*, *c* (Å)	5.6351 (5), 10.5372 (9), 22.7988 (19)
β (°)	93.696 (1)
*V* (Å^3^)	1350.9 (2)
*Z*	2
Radiation type	Mo *K*α
μ (mm^−1^)	0.20
Crystal size (mm)	0.35 × 0.17 × 0.11

Data collection	
Diffractometer	Bruker APEXII CCD
Absorption correction	Multi-scan (*SADABS*; Krause et al., 2015 ▸)
*T*_min_, *T*_max_	0.93, 0.98
No. of measured, independent and observed [*I* > 2σ(*I*)] reflections	23407, 3594, 3145
*R*_int_	0.025
(sin θ/λ)_max_ (Å^−1^)	0.682

Refinement	
*R*[*F*^2^ > 2σ(*F*^2^)], *wR*(*F*^2^), *S*	0.038, 0.102, 1.04
No. of reflections	3594
No. of parameters	181
H-atom treatment	H-atom parameters constrained
Δρ_max_, Δρ_min_ (e Å^−3^)	0.41, −0.28

Computer programs: *APEX2* (Bruker, 2014 ▸, *SAINT* (Bruker, 2013 ▸), *SHELXT* (Sheldrick, 2015*a* ▸), *SHELXL* (Sheldrick, 2015*b* ▸), *XP* in *SHELXTL* (Sheldrick, 2008 ▸) and *publCIF* (Westrip, 2010 ▸).

## full crystallographic data

### 1,2-Bis[di(benzofuran-2-yl)phosphanyl]ethane Crystal data

**Table:**

C_34_H_24_O_4_P_2_	*F*(000) = 580
*M**_r_* = 558.47	*D*_x_ = 1.373 Mg m^−^^3^
Monoclinic, *P*2_1_/*n*	Mo *K*α radiation, λ = 0.71073 Å
*a* = 5.6351 (5) Å	Cell parameters from 9822 reflections
*b* = 10.5372 (9) Å	θ = 2.6–29.2°
*c* = 22.7988 (19) Å	µ = 0.20 mm^−^^1^
β = 93.696 (1)°	*T* = 150 K
*V* = 1350.9 (2) Å^3^	Needle, colorless
*Z* = 2	0.35 × 0.17 × 0.11 mm

### 1,2-Bis[di(benzofuran-2-yl)phosphanyl]ethane Data collection

**Table:**

Bruker APEXII CCD diffractometer	3594 independent reflections
Radiation source: fine-focus sealed tube	3145 reflections with *I* > 2σ(*I*)
Detector resolution: 8.3333 pixels mm^-1^	*R*_int_ = 0.025
φ and ω scans	θ_max_ = 29.0°, θ_min_ = 1.8°
Absorption correction: multi-scan (SADABS; Krause et al., 2015)	*h* = −7→7
*T*_min_ = 0.93, *T*_max_ = 0.98	*k* = −14→14
23407 measured reflections	*l* = −31→31

### 1,2-Bis[di(benzofuran-2-yl)phosphanyl]ethane Refinement

**Table:**

Refinement on *F*^2^	0 restraints
Least-squares matrix: full	Hydrogen site location: inferred from neighbouring sites
*R*[*F*^2^ > 2σ(*F*^2^)] = 0.038	H-atom parameters constrained
*wR*(*F*^2^) = 0.102	*w* = 1/[σ^2^(*F*_o_^2^) + (0.0496*P*)^2^ + 0.6701*P*] where *P* = (*F*_o_^2^ + 2*F*_c_^2^)/3
*S* = 1.04	(Δ/σ)_max_ = 0.001
3594 reflections	Δρ_max_ = 0.41 e Å^−^^3^
181 parameters	Δρ_min_ = −0.28 e Å^−^^3^

### 1,2-Bis[di(benzofuran-2-yl)phosphanyl]ethane Special details

**Table:**

Geometry. All esds (except the esd in the dihedral angle between two l.s. planes) are estimated using the full covariance matrix. The cell esds are taken into account individually in the estimation of esds in distances, angles and torsion angles; correlations between esds in cell parameters are only used when they are defined by crystal symmetry. An approximate (isotropic) treatment of cell esds is used for estimating esds involving l.s. planes.

### 1,2-Bis[di(benzofuran-2-yl)phosphanyl]ethane Fractional atomic coordinates and isotropic or equivalent isotropic displacement parameters (Å^2^)

**Table:**

	*x*	*y*	*z*	*U*_iso_*/*U*_eq_	
P1	0.71188 (6)	0.84518 (3)	0.46075 (2)	0.02309 (10)	
O1	0.49580 (18)	0.91096 (10)	0.35215 (4)	0.0306 (2)	
O2	0.66914 (17)	0.61479 (9)	0.40832 (4)	0.0265 (2)	
C1	0.4685 (2)	0.92984 (11)	0.49513 (6)	0.0236 (2)	
H1A	0.320524	0.923062	0.469453	0.028*	
H1B	0.439854	0.889731	0.533297	0.028*	
C2	0.6973 (3)	0.92132 (12)	0.38979 (6)	0.0275 (3)	
C3	0.8541 (3)	1.00332 (13)	0.36809 (6)	0.0280 (3)	
H3	1.005756	1.025137	0.385828	0.034*	
C4	0.7503 (3)	1.05138 (13)	0.31355 (6)	0.0273 (3)	
C5	0.8159 (3)	1.14240 (15)	0.27287 (7)	0.0362 (3)	
H5	0.963404	1.185957	0.277924	0.043*	
C6	0.6589 (4)	1.16671 (17)	0.22517 (7)	0.0458 (4)	
H6	0.698030	1.229013	0.197263	0.055*	
C7	0.4426 (4)	1.1010 (2)	0.21721 (7)	0.0491 (4)	
H7	0.339642	1.118676	0.183556	0.059*	
C8	0.3754 (3)	1.01094 (17)	0.25715 (8)	0.0422 (4)	
H8	0.229594	0.965882	0.251760	0.051*	
C9	0.5312 (3)	0.99049 (13)	0.30494 (6)	0.0282 (3)	
C10	0.5509 (2)	0.70025 (12)	0.44235 (5)	0.0227 (2)	
C11	0.3408 (2)	0.65268 (12)	0.45784 (6)	0.0239 (3)	
H11	0.229288	0.693672	0.480968	0.029*	
C12	0.3194 (2)	0.52756 (12)	0.43237 (5)	0.0231 (2)	
C13	0.1490 (3)	0.43112 (13)	0.43126 (6)	0.0284 (3)	
H13	0.008549	0.439902	0.451813	0.034*	
C14	0.1906 (3)	0.32213 (13)	0.39931 (7)	0.0326 (3)	
H14	0.077800	0.255057	0.398549	0.039*	
C15	0.3946 (3)	0.30903 (14)	0.36829 (7)	0.0347 (3)	
H15	0.416334	0.233660	0.346410	0.042*	
C16	0.5668 (3)	0.40349 (14)	0.36860 (7)	0.0327 (3)	
H16	0.705679	0.395315	0.347393	0.039*	
C17	0.5236 (2)	0.51025 (12)	0.40171 (6)	0.0245 (3)	

### 1,2-Bis[di(benzofuran-2-yl)phosphanyl]ethane Atomic displacement parameters (Å^2^)

**Table:**

	*U*^11^	*U*^22^	*U*^33^	*U*^12^	*U*^13^	*U*^23^
P1	0.02388 (17)	0.01797 (16)	0.02763 (18)	−0.00108 (11)	0.00324 (12)	−0.00143 (11)
O1	0.0314 (5)	0.0278 (5)	0.0333 (5)	−0.0038 (4)	0.0058 (4)	0.0004 (4)
O2	0.0255 (5)	0.0207 (4)	0.0339 (5)	0.0000 (4)	0.0059 (4)	−0.0060 (4)
C1	0.0272 (6)	0.0181 (5)	0.0259 (6)	−0.0009 (5)	0.0047 (5)	−0.0026 (5)
C2	0.0349 (7)	0.0203 (6)	0.0281 (6)	−0.0004 (5)	0.0073 (5)	−0.0034 (5)
C3	0.0339 (7)	0.0230 (6)	0.0273 (6)	−0.0057 (5)	0.0037 (5)	−0.0013 (5)
C4	0.0327 (7)	0.0240 (6)	0.0259 (6)	−0.0005 (5)	0.0072 (5)	−0.0028 (5)
C5	0.0399 (8)	0.0335 (8)	0.0367 (8)	−0.0043 (6)	0.0135 (6)	0.0027 (6)
C6	0.0671 (12)	0.0451 (9)	0.0267 (7)	0.0055 (8)	0.0143 (7)	0.0072 (7)
C7	0.0620 (12)	0.0565 (11)	0.0275 (7)	0.0084 (9)	−0.0089 (7)	−0.0055 (7)
C8	0.0405 (9)	0.0430 (9)	0.0419 (8)	−0.0020 (7)	−0.0059 (7)	−0.0096 (7)
C9	0.0317 (7)	0.0240 (6)	0.0296 (6)	−0.0012 (5)	0.0086 (5)	−0.0043 (5)
C10	0.0263 (6)	0.0172 (5)	0.0247 (6)	0.0020 (4)	0.0028 (5)	−0.0010 (4)
C11	0.0268 (6)	0.0186 (5)	0.0266 (6)	0.0008 (5)	0.0046 (5)	−0.0012 (4)
C12	0.0268 (6)	0.0188 (5)	0.0234 (6)	0.0015 (5)	−0.0017 (5)	0.0010 (4)
C13	0.0296 (7)	0.0239 (6)	0.0315 (7)	−0.0030 (5)	0.0006 (5)	0.0016 (5)
C14	0.0357 (7)	0.0211 (6)	0.0397 (8)	−0.0050 (5)	−0.0074 (6)	−0.0011 (5)
C15	0.0374 (8)	0.0231 (6)	0.0422 (8)	0.0044 (6)	−0.0077 (6)	−0.0110 (6)
C16	0.0299 (7)	0.0284 (7)	0.0396 (8)	0.0051 (5)	0.0003 (6)	−0.0103 (6)
C17	0.0248 (6)	0.0196 (6)	0.0288 (6)	0.0012 (5)	−0.0014 (5)	−0.0017 (5)

### 1,2-Bis[di(benzofuran-2-yl)phosphanyl]ethane Geometric parameters (Å, º)

**Table:**

P1—C2	1.8028 (14)	C6—H6	0.9500
P1—C10	1.8117 (13)	C7—C8	1.384 (3)
P1—C1	1.8519 (13)	C7—H7	0.9500
O1—C2	1.3829 (18)	C8—C9	1.371 (2)
O1—C9	1.3887 (17)	C8—H8	0.9500
O2—C17	1.3758 (15)	C10—C11	1.3531 (18)
O2—C10	1.3869 (15)	C11—C12	1.4425 (17)
C1—C1^i^	1.533 (2)	C11—H11	0.9500
C1—H1A	0.9900	C12—C17	1.3965 (19)
C1—H1B	0.9900	C12—C13	1.3971 (18)
C2—C3	1.3520 (19)	C13—C14	1.388 (2)
C3—C4	1.4321 (19)	C13—H13	0.9500
C3—H3	0.9500	C14—C15	1.394 (2)
C4—C9	1.394 (2)	C14—H14	0.9500
C4—C5	1.400 (2)	C15—C16	1.390 (2)
C5—C6	1.381 (2)	C15—H15	0.9500
C5—H5	0.9500	C16—C17	1.3850 (18)
C6—C7	1.404 (3)	C16—H16	0.9500
			
C2—P1—C10	100.04 (6)	C9—C8—H8	121.9
C2—P1—C1	99.98 (6)	C7—C8—H8	121.9
C10—P1—C1	97.50 (6)	C8—C9—O1	126.45 (14)
C2—O1—C9	106.27 (11)	C8—C9—C4	123.73 (14)
C17—O2—C10	106.06 (10)	O1—C9—C4	109.80 (12)
C1^i^—C1—P1	110.79 (12)	C11—C10—O2	111.43 (11)
C1^i^—C1—H1A	109.5	C11—C10—P1	133.26 (10)
P1—C1—H1A	109.5	O2—C10—P1	115.17 (9)
C1^i^—C1—H1B	109.5	C10—C11—C12	106.59 (11)
P1—C1—H1B	109.5	C10—C11—H11	126.7
H1A—C1—H1B	108.1	C12—C11—H11	126.7
C3—C2—O1	110.67 (12)	C17—C12—C13	118.91 (12)
C3—C2—P1	128.31 (11)	C17—C12—C11	105.69 (11)
O1—C2—P1	120.64 (10)	C13—C12—C11	135.38 (13)
C2—C3—C4	107.70 (13)	C14—C13—C12	118.18 (14)
C2—C3—H3	126.1	C14—C13—H13	120.9
C4—C3—H3	126.1	C12—C13—H13	120.9
C9—C4—C5	119.42 (14)	C13—C14—C15	121.37 (13)
C9—C4—C3	105.53 (12)	C13—C14—H14	119.3
C5—C4—C3	134.98 (14)	C15—C14—H14	119.3
C6—C5—C4	117.75 (15)	C16—C15—C14	121.64 (13)
C6—C5—H5	121.1	C16—C15—H15	119.2
C4—C5—H5	121.1	C14—C15—H15	119.2
C5—C6—C7	121.14 (16)	C17—C16—C15	115.93 (14)
C5—C6—H6	119.4	C17—C16—H16	122.0
C7—C6—H6	119.4	C15—C16—H16	122.0
C8—C7—C6	121.67 (16)	O2—C17—C16	125.85 (13)
C8—C7—H7	119.2	O2—C17—C12	110.19 (11)
C6—C7—H7	119.2	C16—C17—C12	123.94 (13)
C9—C8—C7	116.23 (16)		
			
C2—P1—C1—C1^i^	−66.72 (13)	C3—C4—C9—O1	1.48 (15)
C10—P1—C1—C1^i^	−168.36 (12)	C17—O2—C10—C11	0.95 (14)
C9—O1—C2—C3	0.12 (15)	C17—O2—C10—P1	177.25 (9)
C9—O1—C2—P1	173.64 (9)	C2—P1—C10—C11	−113.82 (14)
C10—P1—C2—C3	−150.90 (13)	C1—P1—C10—C11	−12.23 (15)
C1—P1—C2—C3	109.56 (13)	C2—P1—C10—O2	70.91 (10)
C10—P1—C2—O1	36.84 (11)	C1—P1—C10—O2	172.49 (9)
C1—P1—C2—O1	−62.70 (11)	O2—C10—C11—C12	0.06 (15)
O1—C2—C3—C4	0.80 (16)	P1—C10—C11—C12	−175.35 (10)
P1—C2—C3—C4	−172.09 (10)	C10—C11—C12—C17	−1.02 (14)
C2—C3—C4—C9	−1.38 (15)	C10—C11—C12—C13	−179.30 (14)
C2—C3—C4—C5	175.51 (16)	C17—C12—C13—C14	−0.27 (19)
C9—C4—C5—C6	−0.7 (2)	C11—C12—C13—C14	177.84 (14)
C3—C4—C5—C6	−177.27 (16)	C12—C13—C14—C15	−0.9 (2)
C4—C5—C6—C7	−1.0 (2)	C13—C14—C15—C16	0.9 (2)
C5—C6—C7—C8	1.2 (3)	C14—C15—C16—C17	0.3 (2)
C6—C7—C8—C9	0.4 (3)	C10—O2—C17—C16	177.01 (13)
C7—C8—C9—O1	175.93 (14)	C10—O2—C17—C12	−1.61 (14)
C7—C8—C9—C4	−2.2 (2)	C15—C16—C17—O2	179.94 (13)
C2—O1—C9—C8	−179.39 (15)	C15—C16—C17—C12	−1.6 (2)
C2—O1—C9—C4	−1.02 (14)	C13—C12—C17—O2	−179.74 (11)
C5—C4—C9—C8	2.4 (2)	C11—C12—C17—O2	1.64 (14)
C3—C4—C9—C8	179.90 (14)	C13—C12—C17—C16	1.6 (2)
C5—C4—C9—O1	−176.00 (12)	C11—C12—C17—C16	−177.01 (13)

Symmetry code: (i) −*x*+1, −*y*+2, −*z*+1.
